# Efficient Adsorption-Assisted Photocatalysis Degradation of Congo Red through Loading ZIF-8 on KI-Doped TiO_2_

**DOI:** 10.3390/ma15082857

**Published:** 2022-04-13

**Authors:** Zhechen Liu, Wanqi Zhang, Xilong Zhao, Xianliang Sheng, Zichu Hu, Qiang Wang, Zhangjing Chen, Sunguo Wang, Xiaotao Zhang, Ximing Wang

**Affiliations:** 1 College of Material Science and Art Design, Inner Mongolia Agricultural University, Hohhot 010018, China; liuzhechen@emails.imau.edu.cn (Z.L.); nmgndcyyzwq@emails.imau.edu.cn (W.Z.); xilonghappy@163.com (X.Z.); 2 College of Science, Inner Mongolia Agricultural University, Hohhot 010018, China; shengxl@iccas.ac.cn (X.S.); huzichu@emails.imau.edu.cn (Z.H.); 2019122144691@emails.imau.edu.cn (Q.W.); 3 Department of Sustainable Biomaterials, Virginia Polytechnic Institute and State University, Blacksburg, VA 24060, USA; chengo@vt.edu; 4 Sungro Bioresource & Bioenergy Technologies Corporation, Edmonton, AB T6R 3J6, Canada; wangsunguo@gmail.com; 5 Inner Mongolia Key Laboratory of Sandy Shrubs Fibrosis and Energy Development and Utilization, Hohhot 010018, China

**Keywords:** ZIF-8, TiO_2_ (KI), photocatalysis, Congo red

## Abstract

Zeolitic imidazolate framework-8 (ZIF-8) was evenly loaded on the surface of TiO_2_ doped with KI, using a solvent synthesis method, in order to produce a ZIF-8@TiO_2_ (KI) adsorption photocatalyst with good adsorption and photocatalytic properties. The samples were characterized by XRD, SEM, EDX, XPS, BET and UV-Vis. The photocatalytic efficiency of the material was obtained by photocatalytic tests. The results indicate that the doping with I inhibited the grain growth and reduced the crystallite size of TiO_2_, reduced the band gap width and improved the utilization rate for light. TiO_2_ (KI) was a single crystal of anatase titanium dioxide. The combination of ZIF-8 and TiO_2_ (KI) improved the specific surface area and increased the reaction site. The ZIF-8@TiO_2_ (KI) for Congo red was investigated to validate its photocatalytic performance. The optimal concentration of Congo red solution was 30 mg/L, and the amount of catalyst was proportional to the degradation efficiency. The degradation efficiency of ZIF-8@TiO_2_ (5%KI) was 76.42%, after being recycled four times.

## 1. Introduction

Dye wastewater mainly comes from the textile, dyeing, paper, leather, coating and dye manufacturing industries [1]. The scale of China’s textile production is the largest in the world, as a major player in the textile industry. A total of 10–15% of dyes escape and enter the water, which is treated and then discharged in the process of textile printing and dyeing [2,3]. Only 1 t of dye of can pollute 20 t of water, and so the degree of pollution and damage caused by dye to water is very large [4]. Long-term contact will damage human organs and lead to nervous system disorders. Most azo dyes can lead to cell mutations, gene mutations and body deformations [5]. The most widely used azo dyes have been proved to cause bladder cancer, spleen tumors and liver cancer [6]. The anionic dye Congo red is widely used in the textile industry and is difficult to be degraded [7,8]. Dye degradation methods include physical, chemical and biological methods [9]. Congo red dye can be degraded by adsorption, as a physical method, and photocatalysis, as a chemical method.

TiO_2_ shows excellent photocatalytic performance and has been widely studied for its good chemical stability, low cost, diverse preparation methods, low harm and high catalytic efficiency [10]. The degradation efficiency of TiO_2_ under UV light is low, and so it requires modification to improve its catalytic performance. Non-metallic N doping in TiO_2_ shows good performance [11]. Previous studies showed that the catalytic performance of halogen-I-doped TiO_2_ is higher than that of N [12]. Iodine has a variety of oxidation states, and the surface charge of the catalyst is easy to change. The doping of iodine can produce more oxygen vacancies, reduce the band gap width and improve the utilization rate of ultraviolet light [13].

MOFs have adjustable channels, a large specific surface area, good stability and unsaturated metal sites [14]. The zeolitic imidazolate framework-8 (ZIF-8) in MOFs is composed of Zn^2+^ or Co^2+^, complexed with N atoms on imidazole ligands to form zeolite-like coordination polymers [15]. ZIF-8 has high thermal and chemical stability, and can maintain its complete structure at high temperature; the porosity of the material also does not change. MOFs@TiO_2_ has a synergistic effect to improve photocatalytic performance by accelerating the transfer of photogenerated electrons [16]. ZIF-8 increases the specific surface area of TiO_2_, which provides a large number of reaction sites for TiO_2_ and inhibits the recombination of electrons and holes.

Although ZIF-8@TiO_2_ has been the focus of many scholars [17,18,19,20,21], ZIF-8@TiO_2_ (KI) has not yet been studied. ZIF-8, KI and TiO_2_ were combined for the first time to degrade Congo red under UV irradiation. We prepared ZIF-8@TiO_2_ (5%KI) for the first time. The degradation performance of Congo red is superior to that of other photocatalytic materials. In this paper, the united technologies of adsorption and photocatalysis were used to catalyze Congo red solution in organic dyes. ZIF-8 material in MOFs with a large specific surface area and strong stability was used as the adsorption material, and TiO_2_ was used as the photocatalytic material. ZIF-8 solved the problems of the small specific surface area and insufficient reaction sites of TiO_2_. Doping TiO_2_ with I improved the photocatalytic degradation efficiency of TiO_2_.

## 2. Materials and Methods

### 2.1. Materials

Potassium iodide, 2-methylimidazole and tetrabutyl titanate were purchased from Macklin (Shanghai, China); N, N-dimethylformamide (DMF), anhydrous ethanol, polyethylene glycol 400 (PEG-400) and Congo red were purchased from Fuchen Chemical (Tianjin, China); glacial acetic acid was purchased from Kemao Chemical (Tianjin, China), and zinc nitrate hexahydrate was purchased from Aike Chemical (Chengdu, China).

### 2.2. Preparation and Testing of Photocatalyst

#### 2.2.1. Preparation of TiO_2_ (KI)

Liquid A: 5 mL glacial acetic acid, 2.5 mL deionized water, 1 mL PEG-400 and potassium iodide (KI) were added to 10 mL absolute ethanol and stirred well. Liquid B: 10 mL tetrabutyl titanate was slowly added to 15 mL absolute ethanol and stirred evenly. Liquid A and liquid B were then stirred vigorously in a magnetic stirrer for 20–30 min at room temperature. Liquid A was slowly dropped into liquid B, which was then stirred vigorously for 2 h after liquid A and liquid B were stirred separately. When the amount of KI increased, the color changed from milky white to light yellow. The sol was sealed in a beaker and stored in a dark place for 24 h to form a soft elastic white solid gel, which was placed in a vacuum drying oven and dried at 100 °C for 24 h to form granular crystals of xerogel. The xerogel was ground into powder in a mortar. The solid particles were ground into a white (low KI) or light yellow (high KI) powder. The finished powder was placed in the crucible, which was placed in a box-type furnace for calcination. The temperature was selected as 350 °C for heating and calcining. The powder in the lower layer was processed through a 200-mesh sieve and then used for the photocatalytic experiment after calcination. The treated TiO_2_ was recorded as TiO_2_ (350 °C).

#### 2.2.2. Preparation of ZIF-8@TiO_2_ (5%KI)

In total, 477.8 mg Zn(NO_3_)_2_6H_2_O, 120 mg 2-methyl imidazole and 0.2 g TiO_2_ (5%KI) were added to 36 mL DMF. The samples were ultrasonicated for 20 min at room temperature. The samples after ultrasound were placed in a 100 mL stainless steel autoclave which was placed in an electric drying oven. The temperature was raised to 140 °C at 5 °C/min and then thermally insulated for 24 h. The temperature was reduced to room temperature at a rate of 0.4 °C/min. The yellow crystalline solids were removed from the inner tank of the stainless-steel autoclave, which was washed with DMF and centrifuged three times, before being dried in a vacuum drying oven at 60 °C for 5 h and ground into powder. For convenience of description, the composite material is represented as ZIF-8@TiO_2_ (5%KI).

#### 2.2.3. Evaluation of Photocatalytic Activity

A catalyst of a certain weight was added to 100 mL Congo red solution, which was placed in a photocatalytic reaction tube. After 30 min of reaction in a dark environment, the adsorption equilibrium between the sample and Congo red solution was achieved, and the influence of adsorption on the photocatalytic effect was avoided [22,23]. After reaction in the dark for 30 min, the mercury lamp was turned on, and 5 mL of the reaction Congo red solution was added to the centrifuge tube every 20 min and centrifuged at a speed of 8000 rpm. The supernatant was taken for a wavelength test at 497 nm. As shown in Figure S1 (in the Supplementary Materials), the maximum absorption wavelength is 497 nm [24]. The photocatalytic efficiency was calculated according to Equation (1).

*η* = 1 − *A_t_*/*A*_0_ (1)

where *η* is the photocatalytic efficiency, *A*_0_ is the initial absorbance value of the sample and *A_t_* is the absorbance value of the solution after photocatalytic time *t*.

The kinetics of the photodegradation was modeled using the first-order kinetics:

*ln*(*A*_0_/*A_t_*) = *ln*(*C*_0_/*C_t_*) = *k*_1_*t* (2)

where *A*_0_ is the initial absorbance value of the sample, *A_t_* is the absorbance value of the solution after photocatalytic time *t*, *k*_1_ (min^−1^) is photodegradation rate constant and t (min) is the photocatalytic time.

### 2.3. Characterization

The crystal morphology and distribution of ZIF-8@TiO_2_ (5%KI) was observed with the FEI 650 scanning electron microscope (FEI Company). The element distribution of ZIF-8@TiO_2_ (5%KI) was observed by EDX test. The morphologies and structures of ZIF-8@TiO_2_ (5%KI) were analyzed by TEM (JEM-2100F, Tokyo, Japan). We used the X-ray photoelectron spectrometer (AXIS-ULTRA DLD, Kratos Company, UK) to analyze the changes in the valence states of the elements. The specific surface area and pore size were analyzed using a V-Sorb 2800TP (Jinepu Technology, Beijing, China). An X-ray diffractometer (XRD-700S) was used to analyze the crystal type of TiO_2_ (KI), ZIF-8 and ZIF-8@TiO_2_ (5%KI). The X-ray source was the Cu target, Kα = 0.15418 nm, the tube voltage was 40 kV, the tube current was 100 mA, the diffraction angle was 2θ and the scanning speed was 10°/min. The crystallite size was calculated and analyzed according to Equation (3) [25].

D = Kλ/(βcosθ) (3)

where K is the Scherrer constant of the diffraction peak (0.89), D is the average crystallite size, λ is the wavelength of the X-ray incident wave, valued at 0.15418 nm, β is the half-peak width of the XRD diffraction peak and θ is the Bragg diffraction angle.

The spectra of different powder samples were measured and characterized by Ultraviolet Diffuse Reflectance Spectroscopy (DRS), which used a Tu-1950 dual-beam UV-Vis spectrophotometer with a scanning range of 200–800 nm. The band gap width was calculated according to Equation (4) [26].

(αhν)^2^ = k(hν − Eg) (4)

where α is the absorption coefficient, h is Planck’s constant, ν is the frequency of the light wave, k is the proportionality constant and Eg is the photon energy (band gap width)

## 3. Results

### 3.1. Crystal Structure Analysis of Photocatalyst

**Figure 1:**
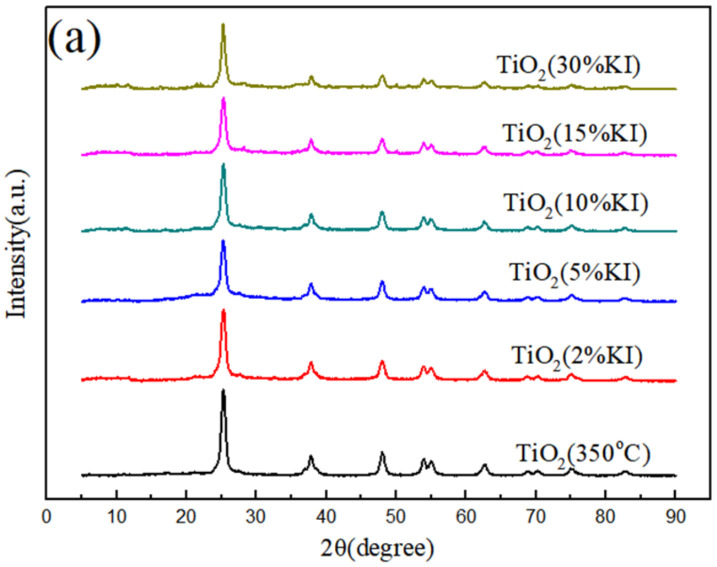

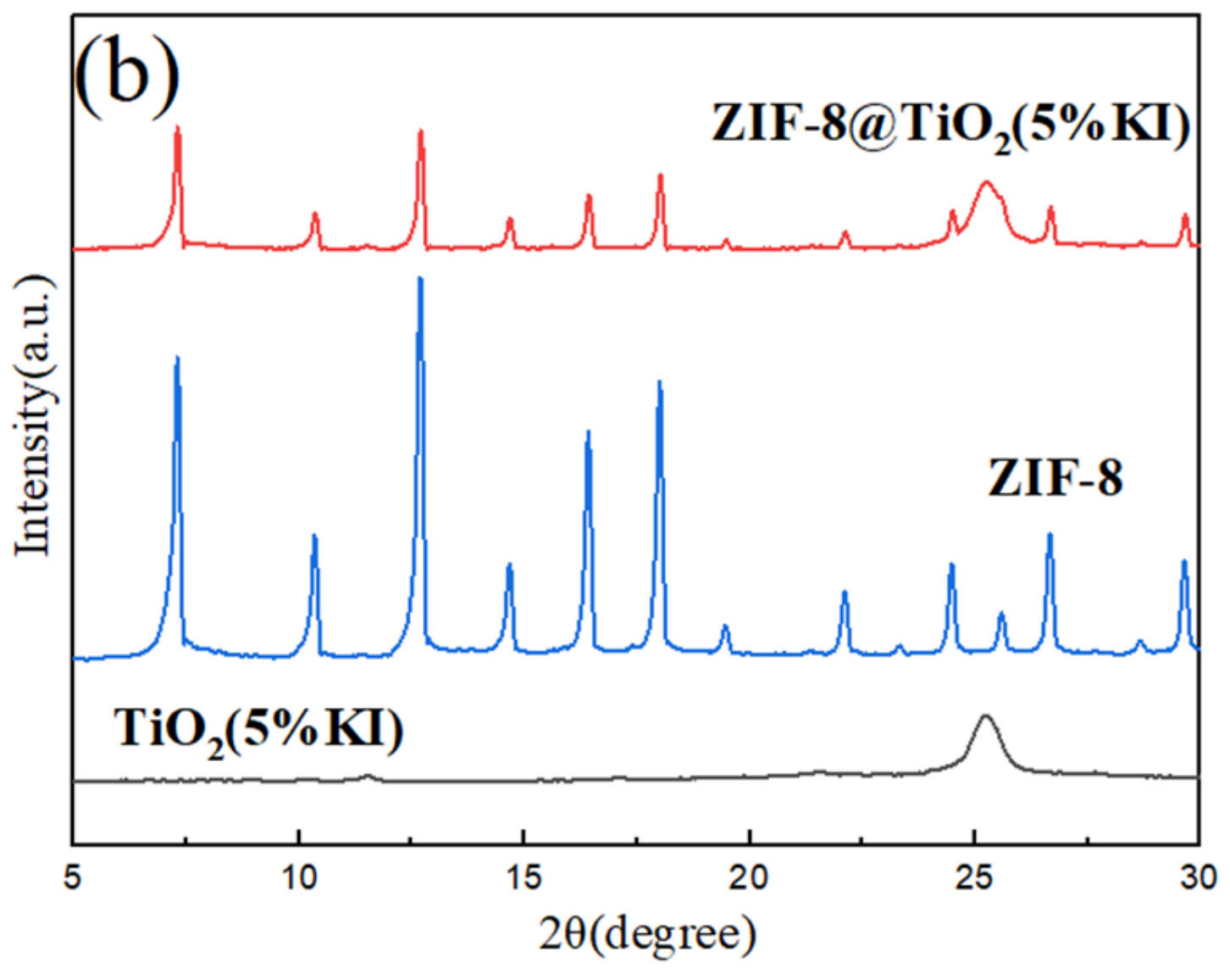
(**a**) XRD patterns of TiO_2_ doped with different amounts of I, (**b**) XRD patterns of TiO_2_ (5%KI), ZIF-8, ZIF-8@TiO_2_ (5%KI).

Figure 1a shows the XRD diagram of potassium-iodide-doped TiO_2_ with different molar ratios and undoped TiO_2_. The calcination temperature of all samples was 350 °C and the calcination time was 5 h. The diffraction angles corresponding to the diffraction peaks in Figure 1 were compared with the standard diffraction card (JCPDS No.21-1272). It was found that the sample was calcined at 350 °C for 5 h to produce a single crystal of anatase-type TiO_2_. There was no rutile-type or plate-type TiO_2_ in the sample. The diffraction angles (2θ) of 25.21°, 37.82°, 47.9° and 62.56° belonged to the crystal planes of (101), (004), (200) and (204) of the anatase type, respectively [27]. It was determined that the prepared TiO_2_ was a single anatase-type TiO_2_ crystal. As shown in Figure 1a, with the increase in KI content, the diffraction peak of 25.21° became wider and less sharp. Doping with I ions led to the lattice distortion of TiO_2_, which weakened the lattice signal and reduced the intensity of the diffraction peak. The XRD test did not detect the related diffraction peak of the iodine compound, indicating that I ions were highly dispersed in TiO_2_.

Figure 1b shows the composite material comparison diagram. It can be seen that the XRD pattern of ZIF-8 presents strong diffraction peaks at 7.35°, 10.34°, 12.68°, 14.66°, 16.5° and 18.0°, corresponding to the crystal planes of (011), (002), (112), (022), (013) and (222), respectively. The position of the diffraction peak was consistent with the standard diffraction card [28]. The crystal plane corresponding to the TiO_2_ (5%KI) diffraction angle (2θ) of 25.21° was an anatase-type (101) crystal plane, as shown in Figure 1b. Due to the different intensities of the ZIF-8@TiO_2_ (5%KI) peaks, individual diffraction peaks are masked.

The crystallite size can be calculated according to Equation (3). As can be seen from Table 1, after the addition of KI to TiO_2_, the crystallite size decreased, which was consistent with the change in XRD peaks.

### 3.2. Morphology Analysis

**Figure 2:**
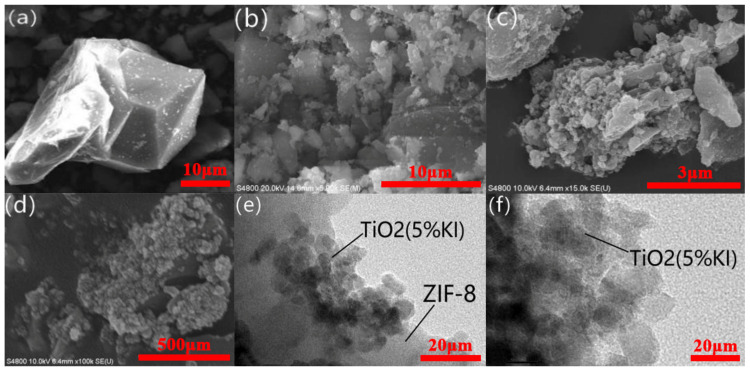
SEM (**a**) of ZIF-8; SEM (**b**–**d**) and TEM (**e**,**f**) of ZIF-8@TiO_2_ (5%KI).

Figure 2a shows the SEM image of ZIF-8. Figure 2b–d show the SEM images of ZIF-8@TiO_2_ (5%KI). Figure 2e,f show the TEM images of ZIF-8@TiO_2_ (5%KI). Figure 2a shows that ZIF-8 was a decahedral sodalite crystal with multiple quadrilateral surfaces [29]. It can be seen from Figure 2b that the material of ZIF-8@TiO_2_ (5%KI) was distributed in a lamellar or block shape. With the increase in the number of SEM tests, the fine distribution of micro-spherical TiO_2_ (5%KI) can be observed in Figure 2c,d. In these images, the bright part is the micro-spherical TiO_2_ (5%KI), and the black part is the block ZIF-8 material under the TiO_2_ (5%KI). The agglomeration of TiO_2_ (5%KI), which was densely distributed around ZIF-8, is shown in Figure 2d. Figure 2e demonstrates the compound situation of ZIF-8@TiO_2_ (5%KI). It can be seen that ZIF-8-coated micro-spherical TiO_2_ (5%KI) had a good coating effect. ZIF-8 was on the outside of the composite, and TiO_2_ (5%KI) was on the inside of the composite. In Figure 2e, the black spherical part in the middle is the encapsulated and agglomerated TiO_2_ (5%KI). Figure 2f provides a magnified view of the agglomeration part of TiO_2_ (5%KI). The agglomeration phenomenon was caused by the uneven distribution of TiO_2_ (5%KI) in the process of preparing ZIF-8@TiO_2_ (5%KI).

### 3.3. Element Analysis

**Figure 3:**
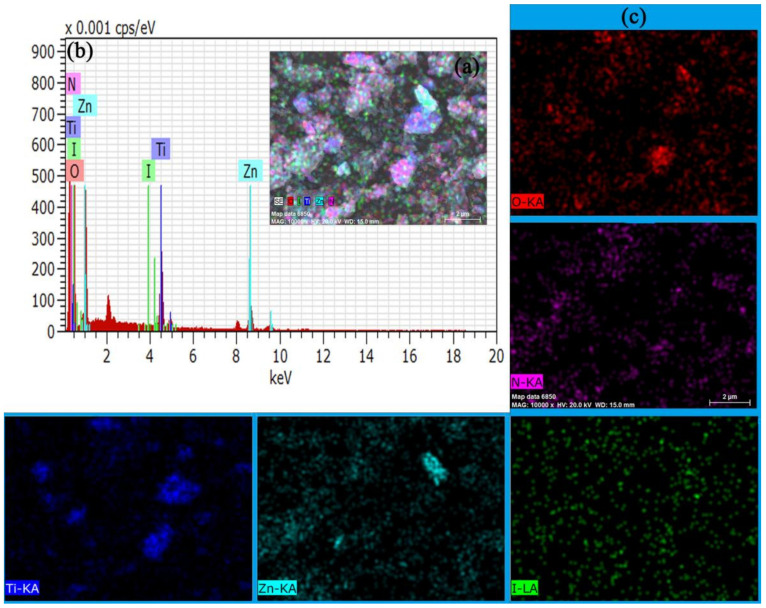
(**a**,**b**) ZIF-8@TiO_2_ (5%KI) EDX diagram, (**c**) single element EDX diagram of ZIF-8@TiO_2_ (5%KI).

Figure 3a,b show the EDX images of ZIF-8@TiO_2_ (5%KI). Figure 3c is the mapping of a single element. The bond between Ti and O was verified by XRD detection. The distribution of I was the same as that of Ti, which indicates that I was doped into TiO_2_ or distributed on the surface of TiO_2_. In XRD detection, the doping of TiO_2_ with I led to the change in crystallite size, so I was doped into TiO_2_ [30]. The distribution of Ti and Zn in the composite material was different, because the distribution of TiO_2_ (5%KI) in the composite material was not uniform. TiO_2_ (5%KI) exhibited the agglomeration phenomenon and some ZIF-8 did not cover TiO_2_ (5%KI). However, the distribution of Ti and Zn was similar at the locations of ZIF-8-coated TiO_2_ (5%KI).

**Figure 4:**
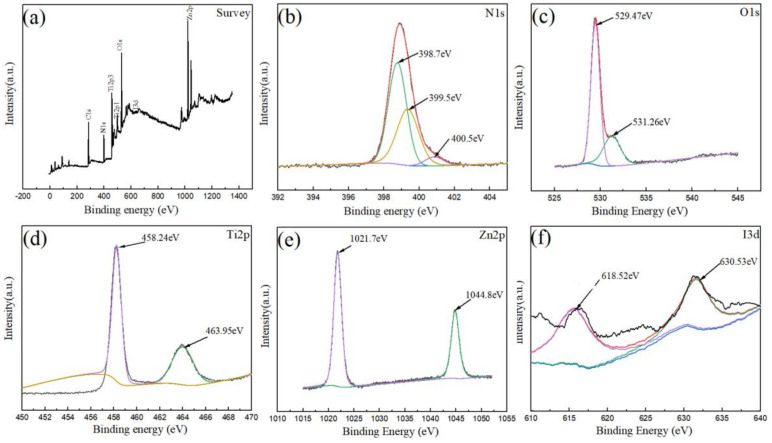
ZIF-8@TiO_2_ (5%KI) XPS analysis diagram. (**a**) XPS diagram of ZIF-8@TiO_2_ (5%KI), (**b**) XPS diagram of N element, (**c**) XPS diagram of O element, (**d**) XPS diagram of Ti element, (**e**) XPS diagram of Zn element, (**f**) XPS diagram of I element.

Figure 4a gives the general diagram of the ZIF-8@TiO_2_ (5%KI) XPS analysis. Nitrogen, oxygen, titanium, zinc, iodine and other elements were present in the composite material. Figure 4b gives the XPS diagram of N in the composite material. N was present in ZIF-8. Three peaks of N appeared in peak fitting, at 398.7 eV, 399.5 eV and 400.5 eV, which correspond to the pyridine-type nitrogen, pyrrole-type nitrogen and graphite-type nitrogen, respectively [31]. Pyridine-type nitrogen and pyrrole-type nitrogen can improve the adsorption performance of ZIF-8 [32,33]. Graphite-type nitrogen can improve the conductivity of ZIF-8. Figure 4c gives the XPS diagram of oxygen. The molecular formula of ZIF-8 is C_8_H_10_N_4_Zn; it was believed that the oxygen present came from the oxygen atom in TiO_2_. The diffraction peaks of oxygen at 529.47 eV and 531.26 eV correspond to lattice oxygen (529.5 eV) and surface-adsorbed oxygen (531.7 eV), respectively [34]. Because of the doping of I, the diffraction peaks of oxygen shifted. The lattice oxygen came from the oxygen in TiO_2_ (Ti-O). It can be inferred that Ti-O-I or oxygen holes are generated [35]. The formation mechanism of oxygen holes means that I^5+^ doped with TiO_2_ generates I_2_O_5_, and the surrounding O and I_2_O_5_ will be further turned into oxygen and low-state I, thus, forming oxygen vacancies on the surface [30]. Adsorbed oxygen existed in the form of a Ti-OH bond. Figure 4d gives the XPS peak fitting diagram of Ti. The diffraction peaks of 458.24 eV and 463.95 eV belong to the Ti 2p_3/2_ and Ti 2p_1/2_ characteristic peaks of Ti, respectively. The characteristic peaks shifted due to the doping with I. Ti existed in the form of +4 valence. Figure 4e gives the XPS peak fitting diagram of Zn. The diffraction peaks of Zn at 1021.7 eV and 1044.8 eV correspond to the characteristic peaks of Zn 2p_2/3_ (1021.3 eV) and Zn 2p_1/2_ (1044.4 eV) [36]. Zn existed in the composite with +2 valence. However, due to the introduction of TiO_2_, the diffraction peak was red-shifted by 0.4 eV [37]. Figure 4f gives the XPS peak fitting diagram of I. The peaks at 618.52 eV and 630.53 eV correspond to the peaks of I 3d_5/2_ and I 3d_3/2_ of I, respectively. It was generally believed that the characteristic peak at 618 eV corresponded to I^−^ and the peak at 630 eV corresponded to I^5+^ [38,39]. Therefore, when I was doped into TiO_2_, the valence state changed from −1 valence in KI to +5 valence, while the rest of the elements did not change their valence state.

### 3.4. Analysis of Specific Surface Area

**Figure 5:**
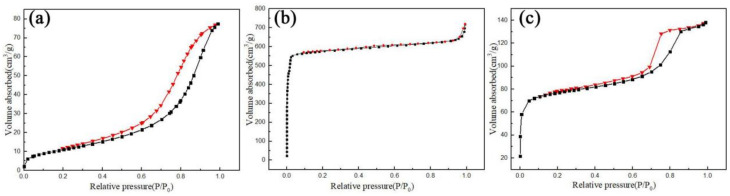
(**a**) N_2_-desorption diagram of TiO_2_ (5%KI), (**b**) N_2_-desorption diagram of ZIF-8, (**c**) N_2_-desorption diagram of ZIF-8@TiO_2_ (5%KI).

Figure 5a gives the N_2_ adsorption desorption diagram of TiO_2_ (5%KI). The N_2_ adsorption–desorption curve of TiO_2_ (5%KI) represents a type IV IUPAC curve [40]. There was a monolayer adsorption of capillary condensation. The curve appeared as a hysteresis loop and the material exhibited the capillary condensation phenomenon when the *P/P*_0_ value was between 0.3 and 1.0. This phenomenon mostly occurs in mesoporous adsorbents. The hysteresis loop belongs to the H3 type and the adsorption amount increases with the increase in pressure. As can be seen from Table 2, the BET-specific surface area of TiO_2_ (5%KI) is 67.233 m^2^/g, the total pore volume (*P/P*_0_ = 0.989) is 0.0976 cm^3^/g, the average pore diameter is 5.8074 nm and the average pore diameter is in the range of the mesopore.

Figure 5b gives the N_2_ adsorption–desorption curve of ZIF-8. The N_2_ adsorption–desorption curve of ZIF-8 represents a type I IUPAC curve, which is a microporous adsorbent with almost no hysteresis loop [40]. As can be seen from Table 2, the BET-specific surface area of ZIF-8 is 1853.02 m^2^/g, the average pore diameter is 2.3243 nm and the total pore volume (*P/P*_0_ = 0.9912) is 1.1119 cm^3^/g.

Figure 5c shows the N_2_ adsorption–desorption curve of ZIF-8@TiO_2_ (5%KI). The N_2_ adsorption–desorption curve of ZIF-8@TiO_2_ (5%KI) represents a type IV IUPAC curve, which is a monolayer adsorption of capillary condensation [40]. When the *P/P*_0_ value is between 0.7 and 0.9, the hysteresis loop appears in the curve. The hysteresis loop belongs to the H2 type, which has a wide hysteresis loop and a steep desorption curve. H2-type hysteresis loops are usually found in porous materials with a wide pore diameter and various pore shapes. As can be seen from Table 2, the BET-specific surface area of ZIF-8@TiO_2_ (5%KI) is 289.92 m^2^/g, the total pore volume (*P/P*_0_ = 0.989) is 0.2135 cm^3^/g, the average pore diameter is 2.9461 nm and the pore type is mesopore.

It can be seen from the comparison in Figure 5a–c that the specific surface area of ZIF-8@TiO_2_ (5%KI) is much larger than that of TiO_2_ and much smaller than that of ZIF-8. Due to the loading of ZIF-8, the specific surface area of ZIF-8@TiO_2_ (5%KI) is greatly improved, but is greatly reduced for ZIF-8. The ultimate goal of the experiment was to obtain a material with a higher specific surface area than TiO_2_ through the composite of ZIF-8 and TiO_2_. Finally, we obtained a photocatalytic material with a high specific surface area.

### 3.5. Analysis of UV-Vis Diffuse Reflectance Spectroscopy (UV-Vis DRS)

**Figure 6:**
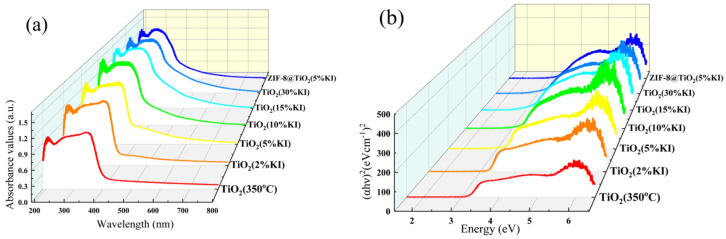
DRS diagram (**a**) and band gap width diagram (**b**).

XRD shows that the crystal of TiO_2_ is of the anatase type. The band gap of anatase-type TiO_2_ was 3.2 eV when TiO_2_ was calcined at 350 °C for 5 h. Figure 6a shows the DRS of TiO_2_ (350 °C), TiO_2_ (KI) and ZIF-8@TiO_2_ (5%KI). Figure 6b provides a plot of the band gap width of the above material. The range of the light response changed, and the UV diffuse reflectance spectrum curve had a red or blue shift after the doping of KI. A red shift occurred in TiO_2_ (5%KI), TiO_2_ (10%KI), TiO_2_ (15%KI) and TiO_2_ (30%KI), which increased the light response range. The blue shift of TiO_2_ (2%KI) and ZIF-8@TiO_2_ (5%KI) reduced the light response range. Table 3 shows the band gap widths of samples. As can be seen from Table 3, the band gap widths of TiO_2_ (5%KI), TiO_2_ (10%KI), TiO_2_ (15%KI) and TiO_2_ (30%KI) decrease in turn, which are all smaller than the band gap of TiO_2_ (350 °C). The reason is that the doping with I causes defects in the TiO_2_ crystal, resulting in defective energy levels, reducing the average particle size of the crystal and increasing internal stress, which led to the overlap of electronic wave function [41]. The blue shift of TiO_2_ (2%KI) and ZIF-8@TiO_2_ (5%KI) was caused by the quantum size effect of the nanoparticles.

### 3.6. Evaluation of Photocatalyst Performance

#### 3.6.1. Effect of KI Doping Amount for Photocatalytic Efficiency

**Figure 7:**
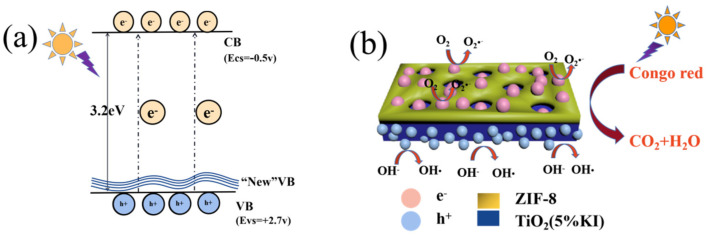
Schematic of the photocatalytic mechanism of (**a**) TiO_2_ (KI) and (**b**) ZIF-8@TiO_2_ (5%KI).

The degradation mechanism of photocatalytic materials is shown in Figure 7. The energy of UV light was greater than the band gap value of anatase TiO_2_ (3.2 eV). Electrons in the TiO_2_ valence band (VB) were excited to the conduction band (CB). Photogenic holes had positive potential in the valence band and photogenic electrons had negative potential in the conduction band. Photogenerated electrons combined with oxygen in the solution to form superoxide radicals (O∙), and photogenerated holes combined with OH^−^ to form hydroxyl radicals (OH∙) [42]. O∙ and OH∙ can mineralize Congo red into small molecules to achieve degradation. The doping of I atoms will generate a new valence band in the gap, which is near the valence band, due to the substitution of Ti^4+^ by I^5+^ [43], and the covalent bond features of I5s, 5P orbitals and O2p [44]. The new valence band makes it easier for photogenerated electrons to transfer to the conduction band, thus, improving the photocatalytic efficiency. ZIF-8 was coated with TiO_2_, and the Congo red in the solution was adsorbed to the surface of ZIF-8. O∙ and OH∙ were transferred to the surface of ZIF-8 via the ZIF-8 channel to degrade the adsorbed Congo red.

**Figure 8:**
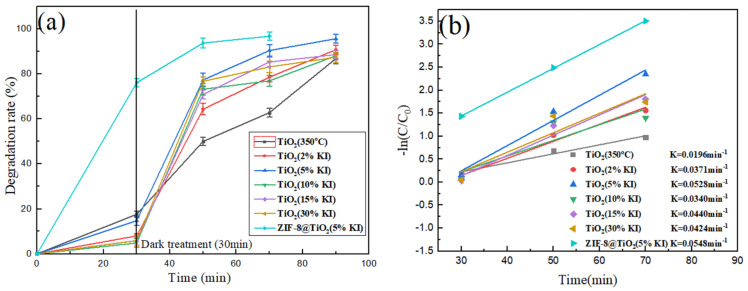

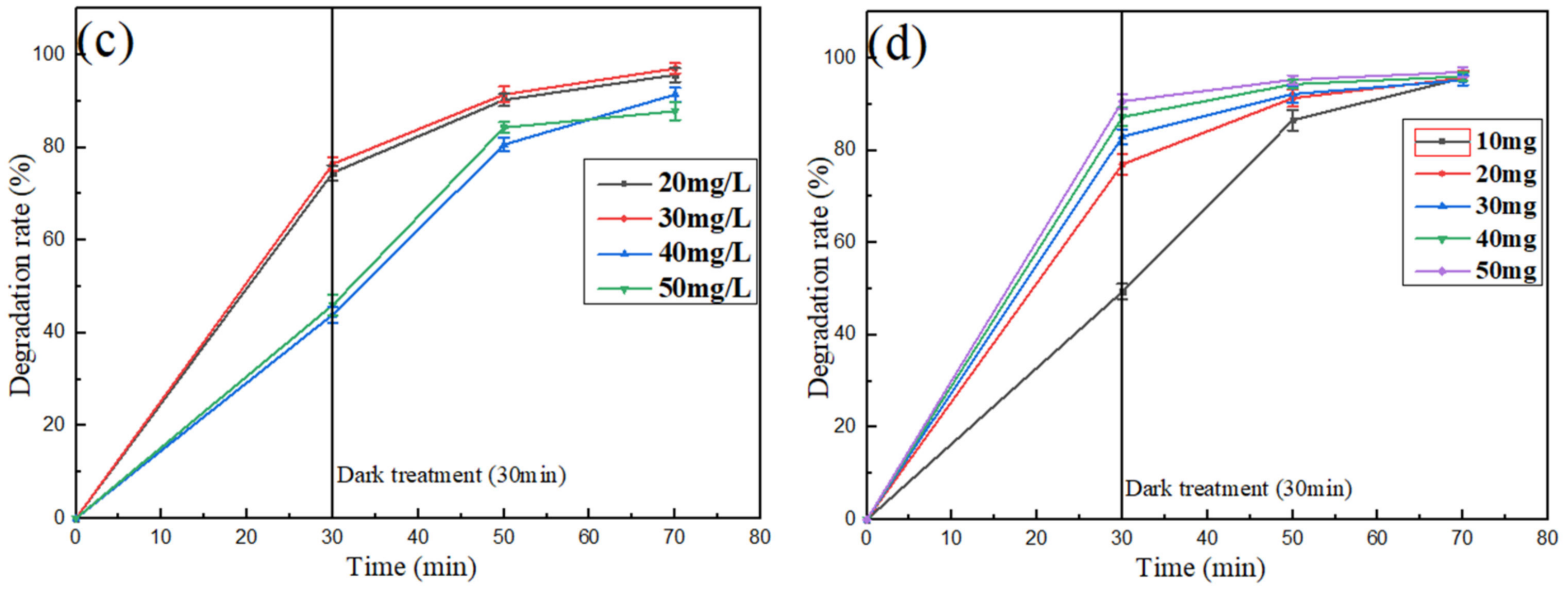
(**a**) ZIF-8@TiO_2_ (5%KI), TiO_2_ (KI) and TiO_2_ (350 °C) photocatalytic efficiency diagram, (**b**) kinetic equation of Congo red degradation, (**c**) effect of the initial concentration of Congo red on catalytic efficiency, and (**d**) effect of catalyst dosage on catalytic efficiency.

The catalysts used were TiO_2_ (350 °C), TiO_2_ (2%KI), TiO_2_ (5%KI), TiO_2_ (10%KI), TiO_2_ (15%KI), TiO_2_ (30%KI) and ZIF-8@TiO_2_ (5%KI). The dosage of the catalyst was 20 mg. A concentration of 20 mg/L and a volume of 100 mL of Congo red solution were selected for the photodegradable material. The test process was the same as that given in 2.2.3, and the photocatalytic efficiency was calculated according to Equation (1). It can be seen from Figure 8a that the photocatalytic efficiency first increased and then decreased in TiO_2_ (350 °C), TiO_2_ (2%KI), TiO_2_ (5%KI), TiO_2_ (10%KI), TiO_2_ (15%KI) and TiO_2_ (30%KI). TiO_2_ (5%KI) had the highest catalytic efficiency. The degradation efficiency of Congo red solution could reach 95.21% after 1 h of UV irradiation. The highest catalytic efficiency found for TiO_2_ (5%KI) was due to the following reasons: The doping of I causes lattice distortion and produces a Ti-O-I bond in TiO_2_ [35]. The I atoms replace Ti atoms to generate a shallow potential trap, which inhibits the recombination of the photo-generated electron and hole [45]. Therefore, the photo-generated electron and hole are further separated, and the photocatalytic efficiency is improved [46]. The light response range of TiO_2_ increased and the phenomenon of red shift occurred after the doping of KI, which improved the utilization rate of ultraviolet light [47].

The doping of I into TiO_2_ results in a reduction in crystal size. With different molar ratios of I doping, the crystallite size of TiO_2_ (5%KI) was the smallest, reaching 14.58 nm. TiO_2_ semiconductors smaller than 16 nm produced quantum size effects [48]. The valence band and conduction band in TiO_2_ became relatively independent of energy levels, the valence band became more corrected, and the conduction band became more negative, which caused the oxidation and reduction ability to become stronger and the photocatalytic efficiency to improve [49]. I^5+^ ions replaced Ti^4+^ ions, which resulted in lattice distortion and a dipole moment [50]. The dipole moment had a positive effect on photoelectron-hole separation. The doping with I not only caused lattice distortion, but also caused the change in the TiO_2_ band gap. The band gap became smaller. The separation of photogenerated electrons and holes became easier, which improved the photocatalytic efficiency with KI doping. Therefore, the photocatalytic efficiency of TiO_2_ can be enhanced by doping KI in TiO_2_. However, with the increase in the KI doping amount, lattice distortion was caused and the crystal structure of TiO_2_ was destroyed. The number of photogenerated electron-hole pairs produced by TiO_2_ was greatly reduced, which reduced the photocatalytic efficiency. Excessive I doping caused the TiO_2_ to generate an excessive shallow potential capture center, which increased the recombination rate of photoinduced electron-hole pairs and reduced the photocatalytic efficiency [51].

The photocatalytic conditions of ZIF-8@TiO_2_ (5%KI) were the same as those of TiO_2_ (5%KI). However, the catalytic efficiency of ZIF-8@TiO_2_ (5%KI) was higher than that of TiO_2_ (5%KI), and the photocatalytic efficiency of ZIF-8@TiO_2_ (5%KI) was 97% under UV irradiation for 40 min. TiO_2_ (5%KI) combined with ZIF-8 greatly improved the catalytic efficiency of TiO_2_ (5%KI), the main reason being that the combination of TiO_2_ (5%KI) and ZIF-8 increased the specific surface area of the material. ZIF-8 provided a large number of reaction sites for the photocatalytic reaction, which solved the problem of insufficient reaction sites for TiO_2_ (5%KI) and improved the photocatalytic efficiency [52].

Figure 8b shows the kinetic equation of Congo red degradation by different catalysts, which conforms to the first-order kinetic equation. The crystallite size is shown in Table 1. The kinetic equation of each material is as follows: TiO_2_ (350 °C) is −*ln*(C/C_0_) = 0.0196x − 0.363, TiO_2_ (2%KI) is −*ln*(C/C_0_) = 0.0371x − 0.962, TiO_2_ (5%KI) is −*ln*(C/C_0_) = 0.0528x − 1.393, TiO_2_ (10%KI) is −*ln*(C/C_0_) = 0.034x − 0.7897, TiO_2_ (15%KI) is −*ln*(C/C_0_) = 0.044x − 1.179, TiO_2_ (30%KI) is −*ln*(C/C_0_) = 0.0424x − 1.042, and ZIF-8@TiO_2_ (5%KI) is −*ln*(C/C_0_) = 0.0548x − 0.1137. It can be seen that the degradation rate constant of TiO_2_ (5%KI) reached 0.0528 min^−1^, and the maximum degradation rate constant of ZIF-8@TiO_2_ (5%KI) reached 0.0548 min^−1^. These results indicate that the degradation effect of the two materials on Congo red is relatively excellent.

#### 3.6.2. Effect of Congo Red Concentration on Photocatalytic Efficiency

The dosage of the catalyst was 20 mg, the concentration of Congo red solution was 20 mg/L, 30 mg/L, 40 mg/L, and 50 mg/L and the solution volume was 100 mL. Figure 8c shows the effect of the initial concentration of Congo red solution on the catalytic efficiency. The catalytic efficiency first increased and then decreased with the increase in solution concentration. The Congo red solution of 30 mg/L had the highest catalytic efficiency. The low concentration of Congo red solution was insufficient to make full use of the reaction site. The reaction sites were fully utilized, which enhanced the photocatalytic efficiency when the concentration of Congo red solution reached 30 mg/L. The light penetration ability decreased, which resulted in a weak light response when the concentration of the solution increased. This reduced the generation rate of photogenerated carriers and the photocatalytic efficiency.

#### 3.6.3. Effect of Dosage of ZIF-8@TiO_2_ (5%KI) on Photocatalytic Efficiency

The catalyst was ZIF-8@TiO_2_ (5%KI). The dosage of the catalyst was 10 mg, 20 mg, 30 mg, 40 mg and 50 mg. The concentration of Congo red solution was 30 mg/L, and the solution volume was 100 mL. The test process was the same as that in 2.2.3 and the photocatalytic efficiency could be calculated according to Equation (1). Figure 8d shows the influence of the amount of catalyst on the catalytic efficiency. The amount of catalyst was positively correlated with photocatalytic efficiency. The reaction sites and photocarriers of the catalyst increased with the increase in the amount of catalyst. Under the same conditions, the composite material produced more superoxide radicals and hydroxyl radicals to participate in the photocatalytic reaction [53].

#### 3.6.4. Reuse

The catalyst was ZIF-8@TiO_2_ (5%KI). The dosage of the catalyst was 20 mg. The concentration of the Congo red solution was 30 mg/L and the solution volume was 100 mL. The degradation efficiency of ZIF-8@TiO_2_ (5%KI) was very high after 30 min of dark reaction, as shown in Figure 8a,c,d. In order to balance the adsorption reaction and photocatalytic reaction of the material and avoid the dominance of one of them in the early stages, the dark reaction was selected for 15 min and the photocatalytic reaction for 45 min. The mercury lamp was turned on and reacted for 45 min after the dark reaction for 15 min. After the reaction, 5 mL of the supernatant was centrifuged at a rotating speed of 8000 rpm to measure the absorbance A. The photocatalytic efficiency was calculated according to Equation (1). The bottom photocatalyst was collected, dried at 100 °C for 2 h and reused. As can be seen from Figure S2 (in the Supplementary Materials), the catalytic efficiency reached 83.41% after the third use and decreased to 76.42% after the fourth use. The effect was better after four cycles [54]. The reason for the decrease in catalytic efficiency was that some of the intermediate products were attached to the catalysts, which occupied the reaction site. It may have also been caused by the loss of catalysts during the processes of centrifugation, washing and drying, which led to the reduction in catalytic efficiency. The former was the dominant factor. The combination of KI and ZIF-8 improved the photocatalytic performance of TiO_2_, which was higher than that of the same type ZIF-8@TiO_2_ photocatalyst [21,55,56,57] and higher than that of the same type of Congo red degradation materials [58,59,60,61,62,63]. As shown in Table 4, the degradation effect of ZIF-8@TiO_2_ (5%KI) showed excellent performance in the same type of materials. Compared with materials of the same type, organic pollutants can be rapidly degraded in a short time. The degradation efficiency of ZIF-8@TiO_2_ (5%KI) was the highest in the shortest time, showing a better performance compared with other materials.

## 4. Conclusions

In this paper, TiO_2_ (KI) was prepared by the sol-gel method, and ZIF-8@TiO_2_ (5%KI) was prepared by the solvothermal synthesis method. When calcined at 350 °C for 5 h, the crystal produced (KI) was anatase-type TiO_2_. The doping with I caused the decrease in crystallite size. The ZIF-8@TiO_2_ (5%KI) microstructure was that of an agglomeration of ZIF-8-coated TiO_2_ (5%KI) and a part of TiO_2_ (5%KI). The composite of ZIF-8 and TiO_2_ (5%KI) increased the specific surface area of TiO_2_ (5%KI). I partially replaced Ti and existed in the form of Ti-O-I in TiO_2_ (KI). The doping with I increased the photoresponse range of TiO_2_ and improved the efficiency of the TiO_2_ catalysis of Congo red under ultraviolet light. TiO_2_ (5%KI) had the highest photocatalytic efficiency. Under UV irradiation, the degradation rate of 20 mg/L and 100 mL Congo red solution by 20 mg TiO_2_ (5%KI) for 1 h was 95.21%. Under UV irradiation, the degradation rate of 20 mg/L and 100 mL Congo red solution by 20 mg ZIF-8@TiO_2_ (5%KI) for 40 min was 97%. The optimal concentration of Congo red solution was 30 mg/L, and the catalytic efficiency was 76.42% after being recycled four times.

## Figures and Tables

**Table 1:** Effect of doping with I on the crystallite size of TiO_2_.

Sample	Half Band Width (β)	Bragg Diffraction Angle (2θ)	Bragg Smi-Diffraction Angle (θ)	Crystallite Size (nm)
TiO_2_ (350 °C)	0.8096	25.27	12.635	16.99
TiO_2_ (2%KI)	0.8758	25.24	12.62	15.69
TiO_2_ (5%KI)	0.9433	25.26	12.63	14.58
TiO_2_ (10%KI)	0.8431	25.26	12.63	16.31
TiO_2_ (15%KI)	0.8508	25.27	12.635	16.17
TiO_2_ (30%KI)	0.8386	25.24	12.62	16.39

**Table 2:** Specific surface area and pore size of TiO_2_ (5%KI), ZIF-8, ZIF-8@TiO_2_ (5%KI).

Sample	SBET (m^2^/g)	Vpore (cm^3^/g)	RAve (nm)
TiO_2_ (5%KI)	67.233	0.0976	5.8074
ZIF-8	1853.02	1.1119	2.3243
ZIF-8@TiO_2_ (5%KI)	289.92	0.2135	2.9461

**Table 3:** Forbidden material band widths.

Sample	TiO_2_ (350 °C)	TiO_2_ (2%KI)	TiO_2_ (5%KI)	TiO_2_ (10%KI)	TiO_2_ (15%KI)	TiO_2_ (30%KI)	ZIF-8@ TiO_2_(5%KI)
Eg(eV)	3.20	3.267	3.150	3.138	2.986	2.970	3.239

**Table 4:** Comparison of photocatalytic properties.

Materials	Concentration	Time (min)	Degradation of Material	Degradation Rate	Ref.
ZIF-8@TiO_2_ (5%KI) (This work)	20 mg/L	40	Congo red	97%	This work
ZIF-8@TiO_2_	-	120	Methylene blue	87.5%	[21]
ZIF-8@TiO_2_	100 mg/L	120	Tetracycline	95%	[55]
ZIF-8@TiO_2_	20 mg/L	180	Methylene blue	99%	[56]
ZIF-8@TiO_2_	5 mg/L	40	Methylene blue	91.2%	[57]
Graphene oxide	10 mg/L	90	Congo red	85%	[58]
ZnO/SnO_2_	5 mg/L	120	Congo red	88.14%	[59]
Graphene based Cr substituted β ZnS	20 mg/L	180	Congo red	84.49%	[60]
Silica@ZnO	20 ppm	140	Congo red	93%	[61]
Cr–ZnO	20 mg/L	60	Congo red	94%	[62]
Bi_2_S_3_	35 mg/L	150	Congo red	98%	[63]

## Data Availability

Data sharing not applicable.

## Supplementary Materials

The following supporting information can be downloaded at: https://www.mdpi.com/article/10.3390/ma15082857/s1, Figure. S1. Wavelength scanning curve of Congo red, Figure. S2. Recycling efficiency of ZIF-8@TiO_2_ (5%KI).
