# Supplementary material for: Efficient Adsorption-Assisted Photocatalysis Degradation of Congo Red through Loading ZIF-8 on KI-Doped TiO2
Source: Materials (Basel). 2022 Apr 13;15(8):2857. doi: 10.3390/ma15082857 (PMC9027197; doi:10.3390/ma15082857)
Supplement: Supplementary file 1 [file materials-15-02857-s001.zip › materials-1660338-supplementary.pdf]

Supporting Information

Efficient Adsorption-assisted Photocatalysis Degradation of Congo red through loading ZIF-8 on KI-doped TiO_2

Zhechen Liu[1, †]; Wanqi Zhang[1, †]; Xilong Zhao[1, †]; Xianliang Sheng[2]; Zichu Hu[2]; Qiang Wang[2]; Zhangjing Chen[3]; Sunguo Wang[4]; Xiaotao Zhang[2,5, *]; Ximing Wang[1,5, *]

[1] College of Material Science and Art Design, Inner Mongolia Agricultural University, Hohhot, 010018, China.

[2] College of Science, Inner Mongolia Agricultural University, Hohhot, 010018, China.

[3] Department of Sustainable Biomaterials, Virginia Polytechnic Institute and State University, Blacksburg, VA, 24060, USA.

[4] Sungro Bioresource & Bioenergy Technologies Corp, Alberta, T6R3J6, Canada.

[5] Inner Mongolia Key Laboratory of Sandy Shrubs Fibrosis and Energy Development and Utilization, Hohhot, 010018, China.

[*] Correspondence should be addressed to Xiaotao Zhang and Ximing Wang.

E-mail: lianzixiaotao@163.com; wangximing@imau.edu.cn.

[†] Contributed equally to this paper.

1. Wavelength scanning curve of Congo red

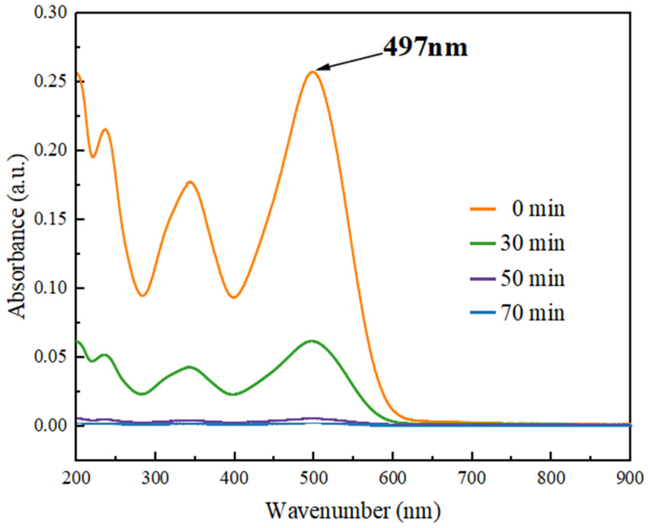

Figure. S1. Wavelength scanning curve of Congo red.

2. *Recycling efficiency of ZIF-8@TiO_2 (5%KI)*

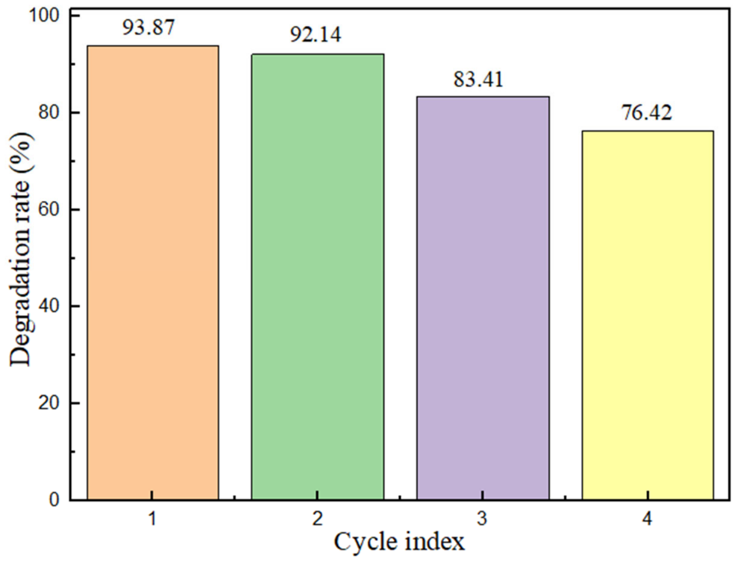

Figure. S2. Recycling efficiency of ZIF-8@TiO_2 (5%KI).
